# Prolonged Survival in Primary Intimal Sarcoma of the Pulmonary Artery: A Rare Case Report

**DOI:** 10.7759/cureus.100292

**Published:** 2025-12-28

**Authors:** Nikoleta Stanitsa, Emmanouil Tempelis, Samiotis Ilias, Orestis Paliaroutas, Panagiotis Dedeilias

**Affiliations:** 1 Cardiothoracic Surgery, Evangelismos General Hospital, Athens, GRC; 2 Cardiac Surgery, Evangelismos General Hospital, Athens, GRC; 3 Orthopedic Surgery, ΚΑΤ Hospital, Athens, GRC

**Keywords:** cardiac surgery, intimal sarcoma, pulmonary mass, rare tumor, s: pulmonary artery sarcoma

## Abstract

Pulmonary artery intimal sarcoma (PAS) is an exceedingly rare malignant tumor that frequently mimics pulmonary embolism, leading to delays in diagnosis and treatment. We report the case of a 41-year-old woman who presented with progressive dyspnea and was initially treated for presumed pulmonary embolism. Advanced imaging revealed features suggestive of a primary pulmonary artery tumor, and she underwent surgical resection, confirming high-grade intimal sarcoma. Despite recurrence four years later requiring repeat surgery and eventual metastatic progression, the patient survived more than five years, substantially longer than typical outcomes reported for this malignancy. This case highlights the importance of early recognition, the diagnostic value of multimodality imaging, and the critical role of aggressive surgical management in improving survival in PAS.

## Introduction

Primary pulmonary artery intimal sarcoma (PAS) is a rare malignant mesenchymal tumor originating from the intimal layer of the pulmonary artery. It was first described in 1923 by Mandelstamm based on autopsy findings [1]. Fewer than 500 cases have been reported, and the condition is often misdiagnosed as pulmonary embolism due to overlapping imaging characteristics and nonspecific clinical symptoms [2]. Patients typically present with dyspnea, chest pain, or signs of right-sided heart strain, delaying accurate diagnosis.

Cross-sectional imaging with computed tomography (CT), magnetic resonance imaging (MRI), and fluorodeoxyglucose positron emission tomography (FDG-PET) improves diagnostic accuracy by identifying features suggestive of neoplastic rather than thromboembolic disease [3].

The prognosis of PAS remains poor, with median survival commonly reported under 18 months; however, complete surgical resection offers the best chance for extended survival [4]. Multimodality therapy, including chemotherapy and repeat surgical intervention, may further improve outcomes in selected cases [5].

We describe a case of PAS in a young woman who survived more than five years through early detection, two major surgical resections, and systemic therapy.

## Case presentation

A 41-year-old woman presented with a six-week history of progressively worsening exertional dyspnea, with significant deterioration noted over the preceding two weeks. She also reported intermittent pleuritic chest pain and a persistent non-productive cough. Her medical history included well-controlled bronchial asthma, with no history of thromboembolic disease, malignancy, recent surgery, or prolonged immobility.

On physical examination, she was mildly tachypneic with an oxygen saturation of 92% on room air. Cardiorespiratory examination revealed decreased breath sounds bilaterally, without wheezing or rales. Baseline laboratory tests, including D-dimer, complete blood count, coagulation profile, and cardiac biomarkers, were within normal limits.

**Figure 1:**
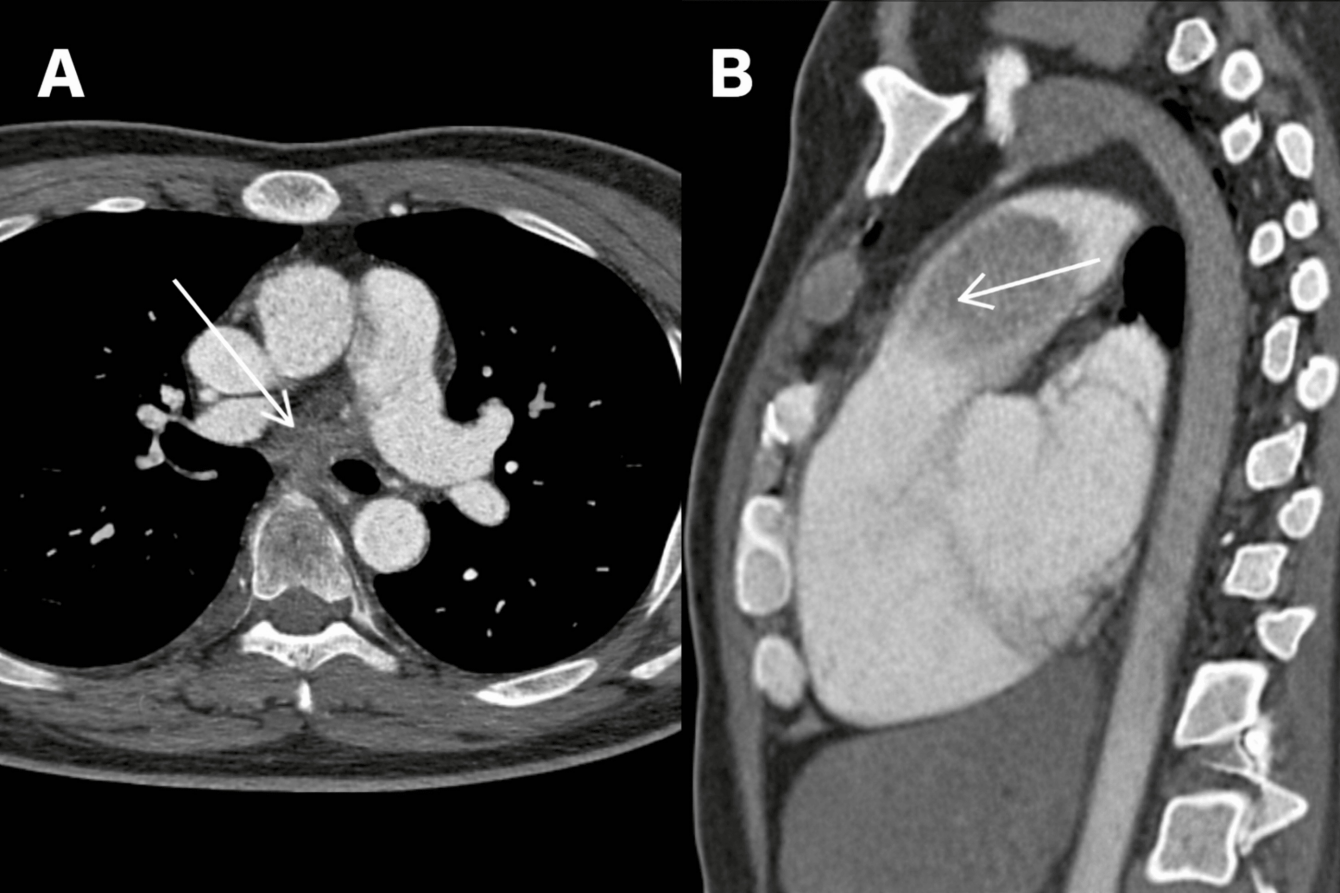
Contrast-enhanced computed tomography pulmonary angiography (A) Axial image showing an intraluminal soft-tissue mass (arrow) in the main pulmonary artery causing near-complete obstruction. The lesion demonstrates a broad-based attachment to the arterial wall, which is atypical for thrombus. (B) Sagittal image again demonstrating the mass (arrow) extending longitudinally along the main pulmonary artery toward the right ventricular outflow tract.

CT pulmonary angiography (CTPA) revealed a large intraluminal filling defect within the main pulmonary artery, measuring 43 × 36 × 37 mm (Figure 1).

A presumptive diagnosis of acute pulmonary embolism was made, and therapeutic anticoagulation with low-molecular-weight heparin was initiated. After 10 days, the patient’s symptoms persisted, and repeat CTPA demonstrated no interval change, with the mass appearing lobulated and attached to the arterial wall.

**Figure 2:**
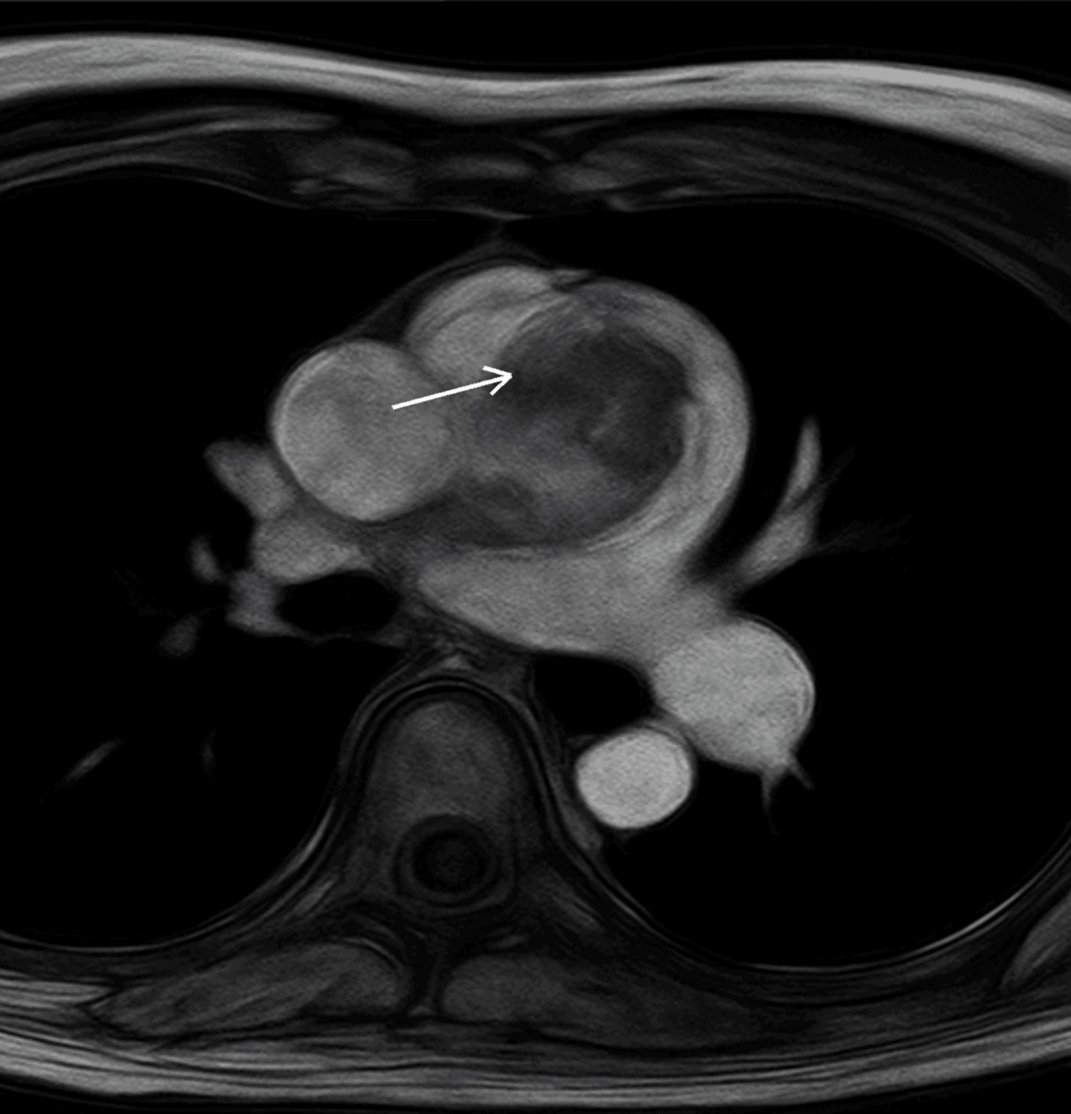
Magnetic resonance imaging of the pulmonary artery mass Axial magnetic resonance imaging demonstrates a large intraluminal soft-tissue mass within the main pulmonary artery with a broad-based attachment to the arterial wall.

MRI revealed a solid, well-defined intraluminal mass demonstrating intermediate T1 and high T2 signal intensity (Figure 2).

FDG-PET demonstrated markedly increased metabolic uptake (SUVmax 17), extending toward the right pulmonary artery and the pulmonary valve.

**Figure 3:**
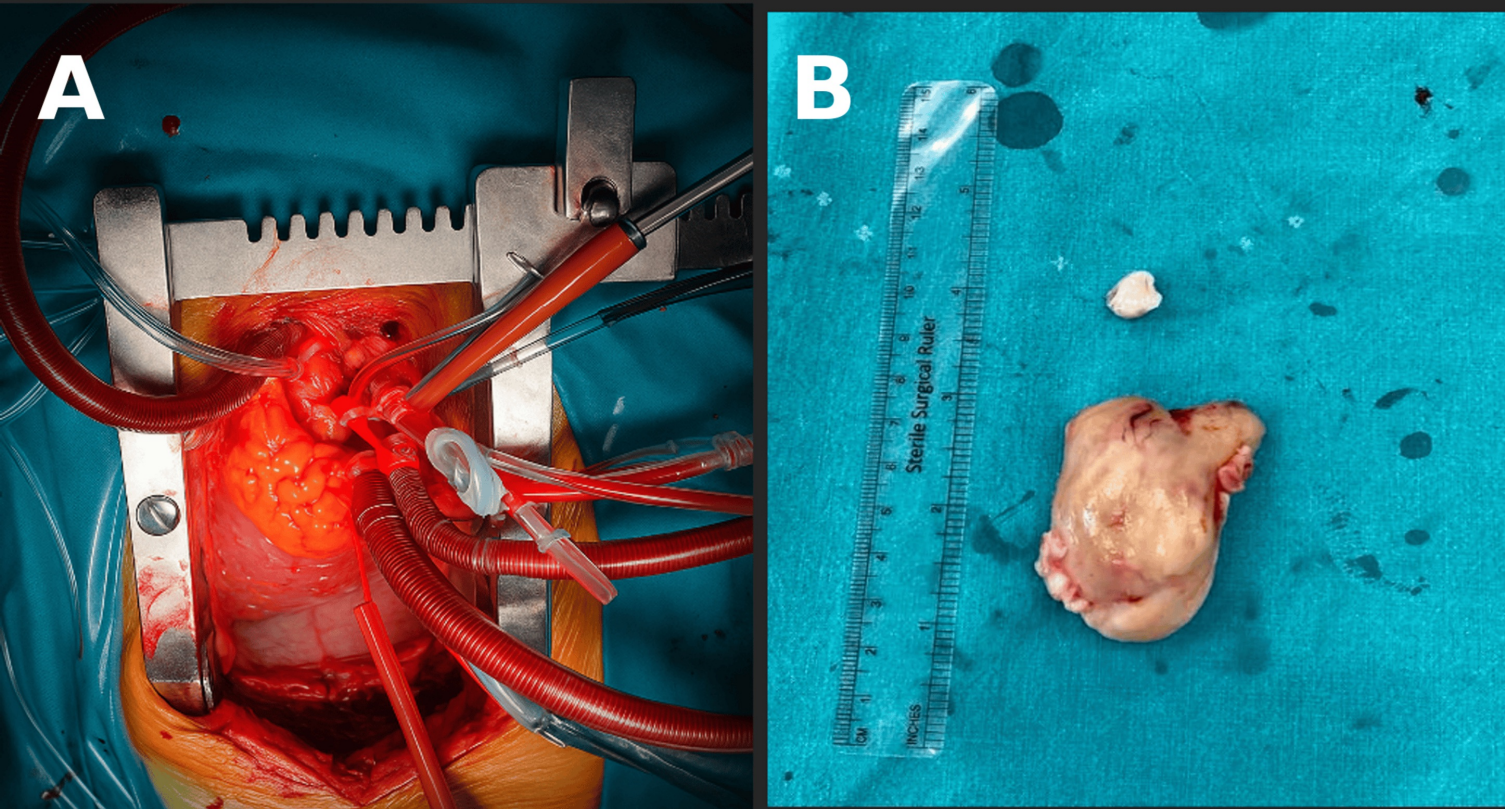
Intraoperative view and resected pulmonary artery sarcoma A) Intraoperative exposure of the main pulmonary artery under cardiopulmonary bypass prior to tumor excision. (B) Gross specimen of the resected mass, a lobulated tan-white intraluminal tumor.

Given the high suspicion of PAS, the patient underwent surgical exploration via median sternotomy. Cardiopulmonary bypass (CPB) was established with aortic and right atrial cannulation under mild hypothermia (32-34°C). The aorta was cross-clamped, and cardioplegic arrest was achieved. Total CPB time was 65 minutes, with an aortic cross-clamp time of 35 minutes. A longitudinal arteriotomy revealed a large polypoid mass originating from the intimal layer of the pulmonary artery. Complete en bloc resection was achieved along with partial arterial wall resection (Figure 3). Frozen section confirmed malignancy.

Histopathologic evaluation demonstrated a high-grade, poorly differentiated intimal sarcoma (FNCLCC grade 3) with extensive necrosis and high mitotic activity.

The postoperative course was uneventful. The patient received adjuvant chemotherapy with doxorubicin and ifosfamide, and later transitioned to gemcitabine and docetaxel due to concerning early postoperative imaging findings. Surveillance imaging over two years showed no recurrence.

**Figure 4:**
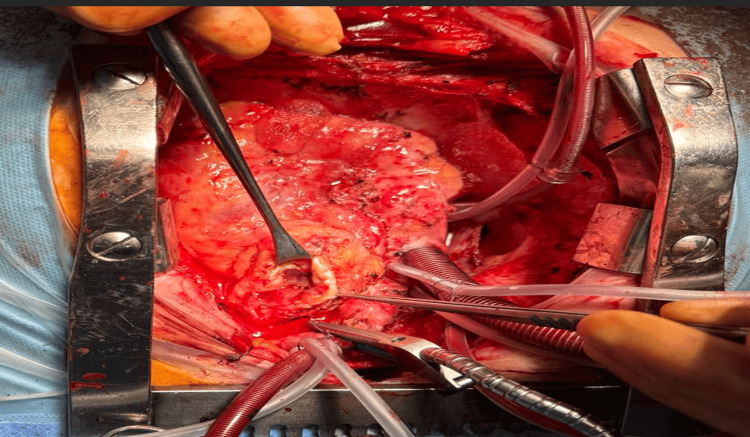
Redo surgery with pulmonary artery opening Intraoperative image from the second operation showing re-entry sternotomy and longitudinal opening of the pulmonary artery under cardiopulmonary bypass. Recurrent intraluminal tumor infiltration is visible, with surgical instruments assisting in debulking.

Four years later, she developed recurrent dyspnea. Imaging revealed tumor recurrence in the pulmonary artery, and she underwent a second median sternotomy with CPB. The aorta was cross-clamped, and cardioplegic arrest was achieved. Total CPB time was 135 minutes, with an aortic cross-clamp time of 95 minutes. Because of extensive arterial invasion, only maximal safe debulking was possible; reconstruction was performed with a bovine pericardial patch (Figure 4), a material widely used for cardiovascular reconstruction, including congenital heart disease procedures [6].

One year later, metastases developed in both lungs, accompanied by severe pulmonary hypertension requiring supplemental oxygen. She was managed with palliative systemic chemotherapy and supportive care. She received an anthracycline-based sarcoma regimen with doxorubicin 75 mg/m² IV on day 1 plus ifosfamide 2.5 g/m²/day IV on days 1-4 (total 10 g/m² per cycle) every 21 days, administered with mesna uroprotection and supportive measures; doses were modified according to tolerance and blood counts.

Despite disease progression, the patient remains alive more than five years after the initial diagnosis.

## Discussion

PAS is an uncommon and highly aggressive neoplasm that poses substantial diagnostic and therapeutic challenges. Its rarity, combined with nonspecific presenting symptoms and radiologic overlap with pulmonary embolism, frequently leads to delayed or incorrect diagnosis. In previously published series, many patients were initially treated for presumed thromboembolic disease before the true nature of their pathology was recognized [2]. Although PAS can often be suspected preoperatively using multiple radiologic modalities (e.g., CT, MRI, and FDG-PET), definitive diagnosis is typically established postoperatively through histopathological examination with immunohistochemical staining; molecular studies such as fluorescent in situ hybridization may be used selectively when needed. This diagnostic delay is clinically significant, as early identification allows for timely surgical intervention, which remains the primary determinant of survival.

Radiologic differentiation between PAS and thromboembolic disease is essential, particularly when patients fail to respond to anticoagulation. Specific CT features-such as a lobulated intraluminal mass, heterogeneous enhancement, and attachment to the arterial wall-may suggest the diagnosis of PAS rather than thromboembolism [3,7]. MRI offers superior soft-tissue characterization and can reveal tumor invasion of the arterial wall or right ventricular outflow tract [8]. FDG-PET provides additional diagnostic value in identifying metabolically active lesions, facilitating the distinction between tumor and chronic thrombus and aiding in the detection of distant metastatic disease [9].

Complete surgical resection has consistently been shown to be the most important factor influencing survival. In the largest available case series, patients who underwent complete resection demonstrated significantly improved survival compared with those who received incomplete resection or palliative therapy [2]. Similarly, long-term institutional experiences emphasize that aggressive surgical management, when feasible, provides the best opportunity for durable outcomes [4]. However, the infiltrative nature of PAS and its predilection for involving the pulmonary artery trunk and valve region often limit the ability to achieve negative margins.

Even after complete resection, recurrence rates remain high, reflecting the biological aggressiveness of PAS. Recurrence may be local, metastatic, or both, and it typically occurs within months to a few years after initial treatment. Selected cases may benefit from repeat surgical intervention, and published reports support the notion that additional resections can contribute to prolonged survival in appropriately selected patients [5,10]. In the present case, the patient remained recurrence-free for several years before requiring a second operation, highlighting the role of vigilant long-term surveillance and the potential value of repeat resection.

Systemic therapy constitutes an important adjunct to surgical management. Given the absence of large prospective trials, treatment strategies are generally extrapolated from data on high-grade soft-tissue sarcomas. Anthracycline-based regimens, particularly doxorubicin in combination with ifosfamide, are considered a standard first-line option [11]. Additionally, gemcitabine-docetaxel therapy has demonstrated activity in recurrent or metastatic soft-tissue sarcomas and is frequently employed in the palliative setting [10]. In this case, both therapeutic strategies were used sequentially, contributing to meaningful periods of disease stability.

This case underscores several important clinical principles. First, persistent pulmonary artery obstruction despite adequate anticoagulation should prompt reconsideration of the initial diagnosis, with particular attention to imaging features suggestive of malignancy. Second, multimodality imaging - including CT, MRI, and FDG-PET - plays a central role in establishing a timely and accurate diagnosis. Third, complete surgical resection remains the cornerstone of therapy and offers the only proven chance for extended survival. Fourth, due to high recurrence rates, long-term surveillance is crucial, and repeat surgical intervention may provide additional survival benefit when technically feasible. Finally, systemic therapy remains an essential component of multidisciplinary care, offering periods of disease control even in the setting of metastatic progression.

## Conclusions

PAS is a rare and highly lethal tumor often mistaken for pulmonary embolism. Early recognition using multimodality imaging allows timely diagnosis and enables potentially curative surgical management. This case demonstrates that long-term survival, exceeding five years, is achievable through aggressive surgical resection, multimodality therapy, and diligent follow-up, even in the presence of recurrence and metastasis.
